# Letter from Dr. Gibbs

**Published:** 1860-01

**Authors:** O. C. Gibbs

**Affiliations:** Frewsburg


### *Letter from Dr. Gibbs.*

FREWSBURG, *December* 12*th*, 1859.

DR. AUSTIN FLINT, JR.:

*Dear Sir.*—In the last No. of your Journal, Dr. J. J. Edmonds, of Buffalo, complains that I misquoted his language in my *Monthly Summary* in the "*American Medical Monthly*," for October. On referring to the papers I find that his complaint is just, and I trust he will please accept my apology. In my life I have never *designed* to misquote or misrepresent another man's opinions; in this case it was wholly unintentional. The preparation of my summary necessitates rather extensive journal reading, some of which is, perhaps, rather hurriedly done. In the haste, I evidently mistook Dr. Edmonds' meaning in one particular, and quoted from memory and incorrectly. The fault in misunderstanding was mine, and not Dr. Edmonds'.

While apologizing for the unintentional misquotation, I have none to offer for the difference in opinion. When we take into consideration the fact that, in perforation of the intestines from disease, the result is almost surely fatal, though, in that case, there may be but one opening, and that, perhaps, quite small, it is not to be wondered at that we should sift the evidence closely, when a man tells us that "there could be no mistake or room for doubt" but that a rifle-ball had passed directly through the intestines—the patient being as well as ever in thirteen days. A ball could not pass, as described by Dr. Edmonds, without making two openings in the peritoneum, and at least two ragged openings in the intestines. When we take into account the course described, and the convoluted condition of the intestines in this region, we should expect that several openings would be made. Through these openings I should expect fecal matter would escape into the cavity of the peritoneum, decomposition of that matter, peritonitis, and death. I should also expect that fecal matter would make its appearance at the external wound, and that when the bowels moved, *evidences of blood* would be discovered in the evacuations. Now, all these conditions were absent, and yet we are gravely told that "there could be no mistake or room for doubt" but the ball "passed through the bowels, wounded the left kidney," &c.

Let Dr. Edmonds wound the intestines of a living or dead animal, and see how long before fecal matter will escape through the lacerated intestine, and then answer to his own judgment if something more positive is not required to prove that, in the case reported, the ball made at least two, and perhaps half a dozen openings in the intestines, and wounded the kidney; and yet the patient was "cheerful" on the second day; had "not much fever and no pain," on the fourth; "pulse. 88, and quite natural," on the eighth; and in condition to discharge on the thirteenth!

Dr. Edmonds thinks the "severe peritonitis" was proof of perforation; and yet, according to his report, the pulse was never above 110. It seems to me that a wound in the peritoneum would fully account for all the symptoms, without seeking for a ball-hole through one or more convolutions of the intestines.

In conclusion, I would say that, as a chronicler of novelties in cases, in opinions, and in practice, I gave expression to my opinions in no captious spirit, or disposition to reflect upon the honor or intelligence of Dr. Edmonds. While I am conscientious in my opinion, differing from his, I must confess that, with the case before him, he had a better opportunity to determine the truth than myself.

Yours truly,

O. C. Gibbs, M. D.

